# Stability, Antioxidant Capacity and Degradation Kinetics of Pelargonidin-3-glucoside Exposed to Ultrasound Power at Low Temperature

**DOI:** 10.3390/molecules21091109

**Published:** 2016-08-24

**Authors:** Jianxia Sun, Zhouxiong Mei, Yajuan Tang, Lijun Ding, Guichuan Jiang, Chi Zhang, Aidong Sun, Weibin Bai

**Affiliations:** 1 Faculty of Chemical Engineering and Light Industry, Guangdong University of Technology, Guangzhou 510006, China; jxsun1220@163.com (J.S.); mzxiong1991@163.com (Z.M.); ddddlj@foxmail.com (L.D.); 18819471250@163.com (C.Z.); 2 Department of Food Science and Engineering, Jinan University, Guangzhou 510632, China; tangyajuan1990@yeah.net; 3 Department of Food Science and Engineering, Shandong Agriculture and Engineering University, Jinan 250199, China; 13705401192@139.com; 4 College of Biological Sciences and Biotechnology, Beijing Forestry University, Beijing 100083, China

**Keywords:** ultrasound, cavitation, degradation, pelargonidin-3-glucoside, antioxidant activity, mechanism

## Abstract

As an alternative preservation method to thermal treatment, ultrasound is a novel non-thermal processing technology that can significantly avoid undesirable nutritional changes. However, recently literature indicated that anthocyanin degradation occurred when high amplitude ultrasound was applied to juice. This work mainly studied the effect of ultrasound on the stability and antioxidant capacity of pelargonidin-3-glucoside (Pg-3-glu) and the correlation between anthocyanin degradation and •OH generation in a simulated system. Results indicated that the spectral intensities of Pg-3-glu decreased with increasing ultrasound power (200–500 W) and treatment time (0–60 min). The degradation trend was consistent with first-order reaction kinetics (R^2^ > 0.9100). Further study showed that there was a good linear correlation between Pg-3-glu degradation and •OH production (R^2^ = 0.8790), which indicated the important role of •OH in the degradation of anthocyanin during ultrasound exposure. Moreover, a decrease in the antioxidant activity of solution(s) containing Pg-3-glu as evaluated by the DPPH and FRAP methods was observed after ultrasound treatment.

## 1. Introduction

Over the past few years, increased fruit and vegetable intake has promoted the development and improvement of new food processing techniques. Traditional thermal processing can cause the loss of nutrients such as vitamin C, carotenoids and flavonoids that are abundant in fruits and vegetables. To maximize the retention of nutrients, many researchers are seeking novel and non-thermal sterilization processing techniques like high hydrostatic pressure (HHP), pulsed electric field (PEF), ultraviolet irradiation, ozone, as well as ultrasound processing [1]. Non-thermal processing can reduce microbial load, and avoid at the same time undesirable changes on food nutrients [2]. Cao et al. [3] found that ultrasound treatment at a frequency of 40 kHz inhibited the decay of strawberry fruit and maintained a significant higher level of vitamin C. Cheng et al. [4] observed that sonicated samples showed better retention or preservation of phenolic compounds when compared to heat-treated samples. However, recent studies have manifested that some non-thermal pasteurization alternatives may lead to the loss of vitamin C, anthocyanins, lycopene, carotenoids and flavonoids under extreme conditions. For example, Yu et al. [5] found that fresh mulberry juice processed by ultra-high pressure at 200 Mpa for 1–3 successive passes suffered a significant reduction of anthocyanins and phenolic acids, as well as ORAC value (*p* < 0.05). The degradation of lycopene was also observed in ultrasound-treated tomato pulp [6]. Valdramidis et al. [7] found that orange juice lost approximately 15% of its ascorbic acid after treatment by ultrasound at the highest amplitude (61.0 μm) and processing temperature (30 °C).

As a kind of important non-thermal food processing technology, ultrasound generally refers to pressure waves with a frequency of 20 kHz or more [1]. As Piyasena et al. [8] pointed out, frequencies from 20 kHz to 10 MHz are used by ultrasound equipment and frequencies between 20 and 100 kHz are called power ultrasound, which can cause cavitation with the generation of free radicals. Advantages of power ultrasound processing include short processing times, minimal thermal effects, higher throughput, and lower energy consumption [9]. The collapse of the cavitation bubble accompanied by the generation of free radicals creates a transitory hot spot, which can dramatically accelerate the chemical reactivity in the medium [10].

Anthocyanins which are abundant in many small berry fruits are relatively unstable and easily susceptible to degradation during processing and storage [11]. The factors affecting anthocyanins include light, oxygen, temperature, pH, structure and concentration of the anthocyanins, and the presence of other compounds, including other flavonoids and phenolics [12]. Among these, previous studies have focused on the thermolysis of anthocyanins. However, with further research in recent years, the adverse impacts of other food processing technology including ultrasound on anthocyanins’ stability are increasingly being recognized. For example, Tiwari et al. [13] found that sonication reduced anthocyanin contents in strawberry juice by 3.2% under the maximum treatment conditions. Chen et al. [14] reported a reduction of anthocyanin extraction yields in raspberries caused by ultrasound, which was explained by chemical reactions that resulted in the degradation of the anthocyanins.

However, to date, there is little knowledge about the degradation behavior and degradation mechanism of monomeric anthocyanins during ultrasonic treatment in simulated systems. Therefore, the purpose of this study was to investigate the effects of ultrasound on anthocyanin stability by a kinetic mathematical model in a model system. The corresponding antioxidant activity changes and the relationship between Pg-3-glu degradation and •OH production was also studied in order to understand the Pg-3-glu degradation mechanim. The monomeric anthocyanin pelargonidin-3-glucoside (Pg-3-glu), one of the major anthocyanins present in strawberry was selected as the research object [15]. The corresponding changes of antioxidant activity and hydroxyl radical generation during ultrasound processing were then determined. Furthermore, the correlation between Pg-3-glu degradation and free radical production induced by ultrasound was analyzed in order to understand the importance of free radicals in the sonochemical degradation of anthocyanins.

## 2. Result and Discussion

### 2.1. Effects of Ultrasound on the UV-Vis Spectra of Pg-3-glu

**Figure 1:**
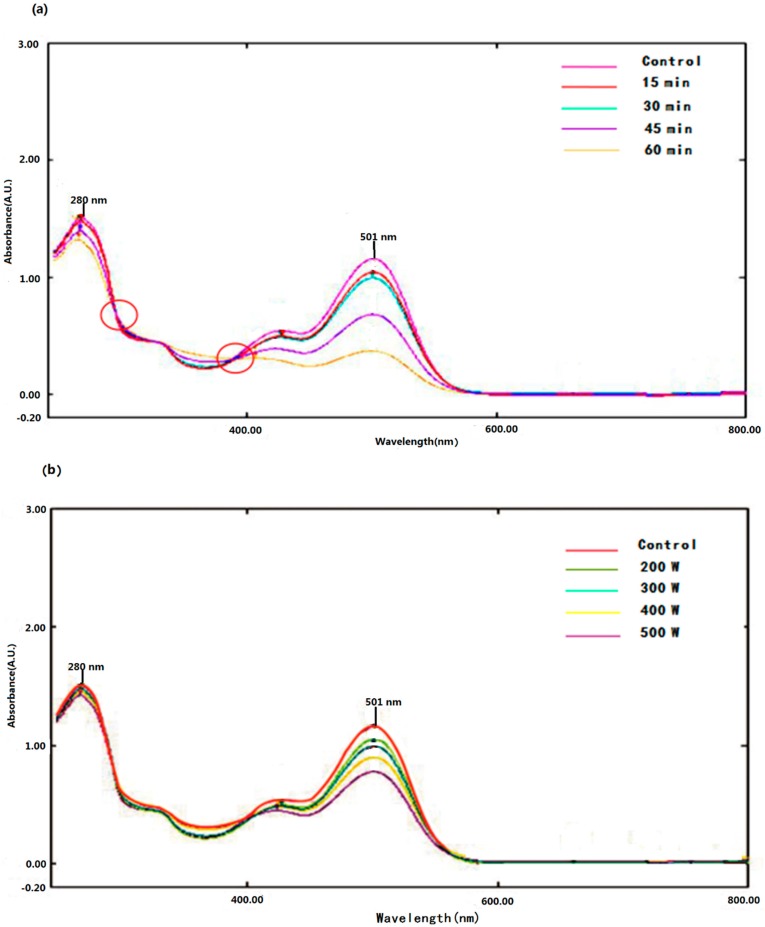
Effect of ultrasonic power and treatment time on the spectra of Pg-3-glu. (**a**) Ultrasonic power 300 W, treatment time varied from 15 min to 60 min; (**b**) treatment time 30 min, ultrasonic power varied from 200 W to 500 W.

The absorption spectrum of Pg-3-glu treated by ultrasound and control are shown in Figure 1. The absorption spectra of control and the treated sample were qualitatively similar, characterized by two sharp peaks at 280 nm and 501 nm and two shoulders at around 335 nm and 430 nm, which was in accordance with earlier reports by Abdel-Aal et al. [16] and Cabrita et al. [17].

In addition, although the spectral pattern of Pg-3-glu was not altered, the spectral intensities of the peaks at 501 nm and 280 nm were decreased after ultrasound treatment, and along with this change, two isosbestic points were formed. This observation is similar to the previously reported spectrum of cyanidin-3-glucoside exposed to a pulsed electric field [18] and delphinidin exposed to thermal degradation [19]. The decay of the absorbance is ultrasonic power and treatment time dependent, which indicated that ultrasound caused the degradation of Pg-3-glu and eventually resulted in an UV-Vis spectrum alteration. 

### 2.2. The Degradation Kinetics of Pg-3-glu Exposed to Ultrasound

Degradation kinetics can be used to predict the rate of health-related compound reduction in juices and nectars during food processing, which is important for controlling food quality. Previous investigations have reported that anthocyanin degradation usually fitted zero-order, first-order, or second-order reaction models. depending on different influencing factors such as heat, H_2_O_2_ and light [20]. However, to date, the degradation kinetics of monomeric anthocyanin exposed to ultrasound treatment have not been reported.

**Figure 2:**
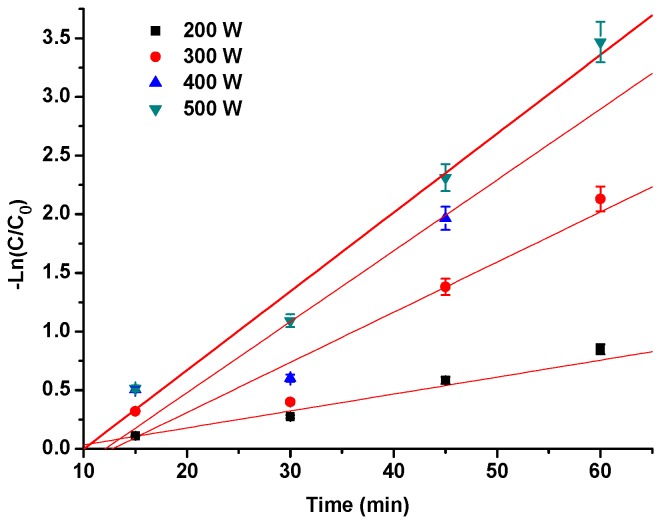
Kinetic analysis of Pg-3-glu during ultrasonic treatment.

The logarithm of the Pg-3-glu contents Ln (C/C_0_) was plotted as a function of time in this study (Figure 2). The linear relationship indicated that the degradation of Pg-3-glu by ultrasound followed first order reaction kinetics (R^2^ > 0.9100). Related kinetics parameters are given in Table 1, which was characterized by the half-life time (t_1/2_) and reaction rate constant (*k*). With increasing ultrasound treatment time and power, the Pg-3-glu degradation amplitude increased significantly (*p* < 0.05). The *k* value ranged from 1.69 × 10^−2^ to 6.72 × 10^−2^ min^−1^ and t_1/2_ ranged from 41.02 to 10.32 min for treatment power in 200–500 W range. Tiwari et al. [13] also reported that at lower ultrasound amplitude levels and treatment times, the anthocyanin levels increased slightly (<1.0%) after ultrasound treatment. However, under the maximum ultrasound treatment conditions, the anthocyanin content in strawberry juice was found to be reduced by 3.2%. They speculated that the increase at the beginning may be explained by the accelerated dissolution of anthocyanins in the suspended pulp. With the increase of the intensity and time of the ultrasound, anthocyanins would degrade due to the cavitation and mechanical action. Tiwari et al. [13] also indicated that a rise in the ultrasound energy increased the cavitation, which can accelerate the degradation of anthocyanins [21].

The thermal degradation reaction rate constants (*k*) of anthocyanin have also been documented in some previous references. A study conducted by Nayak et al. [22] demonstrated that the degradation kinetics of purified anthocyanins followed a first-order reaction with reaction rate constants (*k* values) of 0.0262–0.2855 min^−1^ over the temperature range of 100–150 °C. Mercali et al. [23] reported that the degradation of anthocyanins extracted from acerola pulp could be considered as a first-order model reaction and the rate constants ranged from 5.9 to 19.7 × 10^−3^ min^−1^ with different temperatures from 75 °C to 90 °C. Sui et al. [24] demonstrated the lowest and highest degradation rate constants for cyanidin-3-glucoside were 8.99 × 10^−4^ s^−1^ and 0.120 s^−1^ obtained at pH 2.2 and 100 °C and pH 6.0 and 165 °C, respectively, whereas those for cyanidin-3-rutinoside were 5.33 × 10^−4^ s^−1^ at pH 2.2 and 100 °C and 7.39 × 10^-2^ s^−1^ at pH 5.0 and 165 °C, respectively. The degradation reaction rate constants of cyanidin-3-glucoside exposed to a pulsed electric field (PEF) have also been studied by Zhang et al. [18]. Their data showed that the degradation reaction rate constants ranged from 25.72 s^−1^ to 166.00 s^−1^ at electric field strengths of 7–22 kV/cm.

Compared with the above data, the rate constants of anthocyanin degradation induced by ultrasound in this study were much lower than those of PEF treatment, but were comparable with those of heat treatment at about 100 °C. Taking into account that in this study we used an ice bath to control the temperature rise during the whole ultrasonic treatment process, it can be speculated the main degradation mechanism of anthocyanins induced by ultrasound may be different from thermal degradation. The degradation of Pg-3-glu was mainly attributed to the cavitation effect induced by ultrasound.

### 2.3. Effect of Ultrasound on the Antioxidant Capacity of Pg-3-glu

Some references have reported a change of anthocyanins′ antioxidant activity after heat treatment [25], but as for ultrasound treatment inducing antioxidant activity changes of anthocyanins, it has not been reported prior to present study.

**Figure 3:**
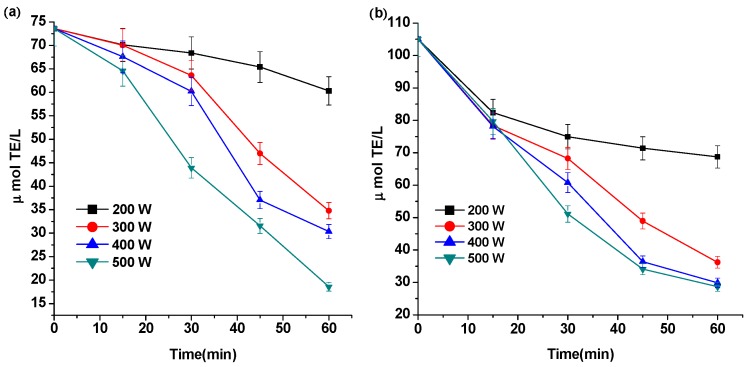
FRAP reducing power (**a**) and DPPH scavenging activity (**b**) changes of solution containing Pg-3-glu after treated by ultrasound. Antioxidant capacity expressed as μmol of trolox equivalents/L sample (μmol∙TE/L).

In this study, the antioxidant activity of control and ultrasound-treated solution containing anthocyanins were evaluated by the FRAP and DPPH methods (Figure 3). The results showed that the antioxidant activity of the solution(s) containing Pg-3-glu decreased with increasing sonication time and/or sonication power. Compared with the control, the highest decrease percentages of the antioxidant activity were 74.77% and 72.74%, obtained at 500 W for 60 min as detected by the FRAP and DPPH methods, respectively.

Anthocyanins are relatively unstable and easily decolorized or degraded because of changes of temperature, pH, oxygen, light, enzymes, hydrogen peroxide, free radicals and so on during food processing and storage, depending on the equilibria between the nine anthocyanin structures [26,27,28].

The above influencing factors may produce different degradation products with different antioxidant activity through different degradation mechanisms and pathways. According to Sun et al., [28] phenolic acids, including 2,4-dihydroxybenzoic, 3,4-dihydroxybenzoic, 2,4,6-trihydroxybenzoic, liberated aglycone, chalcone, and coumarin glucoside are the main thermal degradation products of anthocyanins, and most of them were confirmed to have strong antioxidant and free radical scavenging capacity [29]. Some degradation products even have stronger antioxidant properties than the anthocyanin precursor. For example, a study by Rice-Evans et al. [30] showed that the antioxidant activity of malvidin aglycone is higher than that of malvidin-3-glucoside. Li et al. [31] also found baking anthocyanin-rich purple wheat bran at 177 °C for 20 min increased its DPPH scavenging activity. Yue and Xu [25] reported that the DPPH free radical scavenging capability of bilberry extract increased first and then decreased with increase of heating time when heated at 100 and 125 °C. This was explained by the fact that the degradation products, including phenolic acids and liberated aglycones of anthocyanins, were not stable at high temperature, although they could enhance the antioxidant activity temporarily. Sui et al. [24] found although cyanidin-3-glucoside and cyanidin-3-rutinoside were degraded during heat treatment, the total antioxidant capacity determined by the DPPH assay and ABTS assay was not significantly affected (*p* > 0.05). Another study conducted by Zhang [32] in 2007 found PEF and heat treatment both increased the DPPH free radical scavenging capability and ferric reducing ability of cyanindin-3-glucoside, but decreased the •OH and O_2_•^−^ radical-scavenging abilities.

Compared with the above data, our results show that ultrasound treatment induced a decrease of antioxidant activity of Pg-3-glu, as evaluated by FRAP and DPPH assays in this study, that was different from that of heat and PEF. This may be attributed to the probable different degradation mechanism(s) and degradation products of ultrasound compared with other processing technologies [33]. The literature revealed that ultrasound can initiate various reactions by generating hydroxyl radicals, and enhance polymerization/depolymerization reactions, and improve diffusion rates and other effects [34]. The oxidative degradation products of anthocyanins have also been demonstrated to be different from those of thermal degradation [33]. It remains to be elucidated whether these different antioxidant effects can be ascribed to the variety of degradation products identified by LC-MS. The identification of degradation procucts of Pg-3-glu is ongoing and will be published in the near future.

### 2.4. The Relationship between Reactive Oxygen Specie •OH Generation and Degradation of Pg-3-glu

In order to verify the free radical degradation mechanism of anthocyanins, it is necessary to study the correlation between anthocyanins degradation and •OH generation induced by ultrasound.

**Figure 4:**
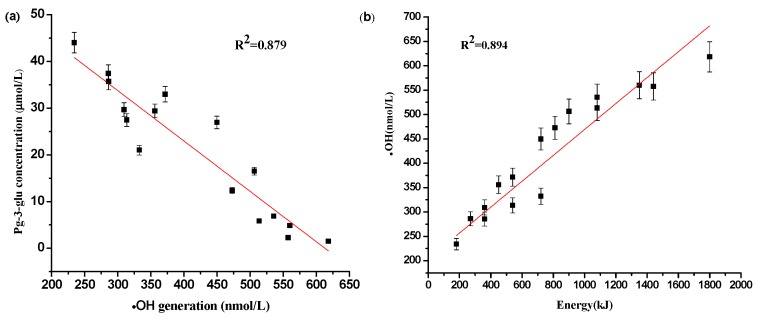
Correlation analysis between (**a**) •OH production and Pg-3-glu concentration and (**b**) total ultrasonic energy output and·OH production.

Results (Figure 4a) showed that there is a good (R^2^ = 0.879) negative correlation between Pg-3-glu concentration and •OH generation, and the latter, measured by monitoring the formation of TA-OH, was well related with the total ultrasonic energy output (R^2^ = 0.894).

This means that the more the •OH production induced by ultrasonic cavitation, the more the Pg-3-glu was degraded, which indicated the important role of •OH in the degradation of anthocyanins. Ultrasonic processing is always accompanied by the occurrence of cavitation which refers to the nucleation formation, growth and implosive collapse of small gas bubbles in liquids, resulting in very high energy densities and in very high local temperatures (up to 5000 K), and local pressures (up to 500 MPa), at the surface of the bubbles for a very short time [10,35]. Under these extreme conditions, several sonochemical reactions occur simultaneously or in isolation. The size and the amount of bubbles formed during ultrasonic processing, the lifetime of the acoustic bubbles and the intensity of its collapse are the main factors in sonolysis degradation [36]. In addition, cavitational thermolysis of water vapor may cleave it into highly reactive species •OH and H•, which can be further followed by the formation of hydroperoxyl radicals and H_2_O_2_. The formation of radicals induced by ultrasonic irradiation is shown in the following Reactions (1)–(4) [37]:

H_2_O → H• + •OH (1)

H• + O_2_ → HO_2_•→•OH + 1/2 O_2_ (2)

2 •OH → H_2_O_2_ (3)

2 HO_2_• → H_2_O + O_2_ (4)

So it can be speculated the degradation of anthocyanins during ultrasonic processing could be induced by the oxidation reactions due to cavitation, promoted by the interaction with free radicals, especially hydroxyl radicals that are formed during the ultrasound treatment. The literature reveals that ROS, including H_2_O_2_, •OH and superoxide anions, can play an important role in the degradation of anthocyanins [38]. Our previous study [28] investigated the oxidation degradation pathways and mechanism of cyanidin-3-sophoroside by PEF. Those results showed that cyaniding-3-sophoroside was degraded through a Baeyer-Villiger oxidation by the nucleophilic attack of hydrogen peroxide and was then further oxidized to the end-product by hydroxyl radical. Ruenroengklin et al. [38] researched the degradation of litchi anthocyanins in the Fenton (Fe^2+^/H_2_O_2_) system. Results showed that •OH radicals induced by the Fenton reaction could increase the anthocyanins’ degradation rate, and the higher concentration of •OH radical had a greater effect on the degradation rate. In fact, a study conducted by K De et al. [39] in 1999 had pointed out that •OH is the main reactive species in the cleavage of the benzene ring in phenolic compounds. However, in order to verify the oxidation degradation assumptions of anthocyanins by ultrasound, identification of the degradations product is necessary in further study.

## 3. Materials and Methods

### 3.1. Chemicals

Pg-3-glu standard for anthocyanins qualitative and quantitative analysis was obtained from Sigma-Aldrich Co.Ltd. (St. Louis, MO, USA). All chemicals in the study were analytical grade, expect that MeOH and methanoic acid were HPLC grade (Aladdin, Shanghai, China). Pure, deionized-distilled water purchased from Watsons (Hongkong, China) was used exclusively in this study.

### 3.2. Extraction and Isolation of Pg-3-glu

Pg-3-glu for experimental materials was extracted from strawberry, which was performed by the modified method described by Sun et al. [40]. Fresh strawberries (Tongzi No.1) were purchased from Beijing Tianyi Strawberry Ecological Park (Beijing, China) and were frozen at −25 °C until use. Then 10 kg of frozen strawberries were thawed overnight (12 h) at 4 °C and mashed by a Joyoung Multi-function Juicer (JYZ-B521, Joyoung Co.,Ltd., Shandong, China). The pulp was extracted with 10 L MeOH contained 0.5% trifluoro acetic acid (TFA, Aladdin, Shanghai, China) at 4 °C for 24 h. The extracts were then filtered on a double layer cheese cloth to remove skin and pulp. Resultant extraction solution was concentrated on a rotary evaporator at 38 °C.

The extracts were then applied on an Amberlite XAD-7 column (70 cm × 2.6 cm, Aladdin, Shanghai, China). After washing the column with 20-fold column volume H_2_O contained 0.5% TFA, the isolated anthocyanins were eluted by methanol containing 0.5% TFA. The elution was evaporated again after being collected by a fraction auto-collector (SBS-100, Shanghai Qingpu Huxi Instrument Co., Shanghai, China). Then 5 mL concentrated isolated methanolic pigments were fractioned by Sephadex LH-20 chromatography (80 cm × 4.5 cm, Pharmacia, Stockholm, Sweden), using H_2_O (0.5% TFA)–MeOH (7:3, *v*/*v*) as eluent. The flow rate was 0.3 mL/min. Fractions were collected by the SBS-100 fraction auto-collector. The purification was carried out at 4 °C. The fractions were evaporated on a rotary evaporator (SENCQ R-501, Shanghai Shenshun Biotechnology Co., Shanghai, China) to remove methanol to facilitate the removal of water remaining in the sample. The evaporation temperature was less than 38 °C. The removal of water was carried out on a freeze drier (LGJ-10, Beijing Songyuan Huaxing Technology Developing Co., Beijing, China).

The individual anthocyanin Pg-3-glu was finally purified using a modified preparative high performance liquid chromatography (HPLC) method, which was carried out with a Venusil ASB-C18 column (25 cm ×2.0 cm; i.d.; 5 mm, Agela, Wilmington, DE, USA) using a BFRL HPLC pump (K-1001, Beijing Rayleigh Analytical Instrument Corporation, Beijing, China) equipped with a UV detector (K-2501, Beijing Rayleigh Analytical Instrument Corporation). Two solvents were used for elution: A = HCO_2_H–H_2_O (5:95; *v*/*v*) and B = HCO_2_H–H_2_O–MeOH (5:45:50; *v*/*v*). The elution profile consisted of an isocratic elution (60% B) for 20 min, linear gradient from 60% to 100% B for 1 min, isocratic elution (100% B) for the next 8 min, followed by a linear gradient from 100% to 60% B for 1 min. The flow rate was 12 mL/min for 30 min and the sample injection volume was 1 mL. Detection was carried out at 280 nm.

### 3.3. Identification and Purity Analysises of Pg-3-glu

The identification of Pg-3-glu was carried out by HPLC/ESI-MS and the purity was analyzed by HPLC-PDA as described by Sun et al. [40]. The purities of Pg-3-glu, which was expressed as the percent area of the isolated anthocyanin at 280 nm, were identified as over 91% at 280 nm and 98% at 520 nm.

### 3.4. Ultrasound Treatment

Ultrasound treatment equipped with a 6 mm diameter probe was performed using an ultrasonic processor (JY92-II DN, Xinzhi Biotech Company, Ningbo, China) with a maximum ultrasound power of 900 W and frequency of 25 kHz. The nominal output power was controlled by setting the amplitude of the sonicator probe, which is tuned to vibrate at a specific frequency, creating pressure waves in the liquid.

Pg-3-glu samples (5 mL) were placed in 10 mL vessels which were cooled in an ice bath during the experimental process (ice was changed every 30 min). Samples were immersed to a depth of 20 mm. Extrinsic parameters output of power (200, 300, 400 and 500 W) and treatment time (15, 30, 45 and 60 min) were designed with pulsed durations of 0.5 s on and 1 s off. The initial temperature of each treated sample is about 4 °C. The final temperature of samples after different ultrasound treatment were listed in Table 2.

### 3.5. Evaluation of •OH Formation

The production of •OH was evaluated by monitoring the formation of hydroxylated terephthalate (TA-OH) between TA and •OH in blank solvent, as described by Freinbichler et al. [41] and Deng et al. [42]. The fluorescence intensity of TA-OH was measured at 342 nm as an excitation wavelength (Ex) and at 440 nm as a emission wavelength (Em) with a Cary Eclipse fluorescence spectrophotometer (Santa Clara, CA, USA). •OH concentration (nM) in the reaction medium was calculated from the standard curve between the fluorescence intensity of TA-OH and the concentration of •OH.

### 3.6. Degradation Kinetics Analysis

The degradation of Pg-3-glu was subjected to the regression analysis using the following first-order models described by Kirca et al. [20]:

ln (C_t_/C_0_) = −*k* × t (5)

t_1/2_ = −ln 0.5 × *k*^−1^ (6)

where C_t_ and C_0_ are the concentration of Pg-3-glu at time t and t_0_, respectively *k* was the reaction rate constant (min^−1^), t was the treatment time (min), t_1/2_ was the half-life degradation values of Pg-3-glu.

### 3.7. Determination of Ultrasonic Energy

The ultrasonic energy was determined from the following equation:

W = P × t(7)

where W is ultrasonic energy (kj), P is the ultrasonic power (W), t is the working time (s).

### 3.8. Methods of Determination

The spectrum of ultrasound-treated Pg-3-glu were recorded on a UV spectrophotometer (UV-1800, Shimadzu Instrument Co. Suzhou, China) at ambient temperature; the scanning wavelength ranged from 250 to 800 nm in steps of 1.00 nm. The quantification of Pg-3-glu was performed by HPLC-DAD (Agilent 1100,) using Pg-3-glu standard according to the method described by Zhang et al. [18], and the initial concentration of Pg-3-glu for experiment material was 49.158 μM. Antioxidant capacity was determined by ferric reducing antioxidant power (FRAP) and 2,2-diphenyl-1-picrylhydrazyl (DPPH) methods. FRAP assay was chosen for evaluation of the total antioxidant activity of bioactive substances. DPPH radical scavenging ability mainly reflects the activity of water-soluble antioxidants. Both FRAP and DPPH assay were using a multimode microplate reader (Infinite F200, Tecan, Mannedorf, Switzerland) according to the procedures described by Aljadi et al. [43] and Du et al. [44] with some modifications, respectively.

### 3.9. Statistical Analysis

All the experiment was performed in triplicate. Statistical analysis of the ANOVA (using Tukey procedure) was conducted using the software Microcal Origin 7.5 (Microcal Software, Inc., Northampton, MA, USA).

## 4. Conclusions

This is the first time the relationship between anthocyanin degradation and hydroxyl radical generation induced by ultrasound in a simulated system was studied. Ultrasound extraction significantly degraded Pg-3-glu, which leads to decreased antioxidant capacity. The degradation of Pg-3-glu exhibited a positive linear relationship with •OH generation, and the degradation kinetics was well fitted to the first-order reaction kinetics. The current results indicate that high intensity ultrasonic treatment should be avoided in engineering design, and it is helpful to optimize and evaluate ultrasound processing conditions for obtaining high-quality berry juices with high levels of bioactives.

## Figures and Tables

**Table 1:** Degradation kinetics parameters of Pg-3-glu exposed to ultrasonic waves.

Parameters	200 W	300 W	400 W	500 W
*k* (min^−1^)	1.69 × 10^−2^	4.28 × 10^−2^	6.50 × 10^−2^	6.72 × 10^−2^
t_1/2_ (min)	41.0146	16.1950	11.4570	10.3147
R^2^	0.9861	0.9211	0.9170	0.9790

**Table 2:** The final temperature of samples after different ultrasound treatment (°C).

Treatment Conditions	200 W	300 W	400 W	500 W
15 min	5.7	10.8	13	17.3
30 min	5.5	10.5	14.6	15.9
45 min	5	8.9	14.6	16.5
60 min	4.8	9.3	14.6	16.1

## References

[1] Gabriel A.A. (2012). Microbial inactivation in cloudy apple juice by multi-frequency dynashock power ultrasound. Ultrason. Sonochem..

[2] Ferrario M., Alzamora S.M., Guerrero S. (2015). Study of the inactivation of spoilage microorganisms in apple juice by pulsed light and ultrasound. Food Microbiol..

[3] Cao S., Hu Z., Pang B., Wang H., Xie H., Wu F. (2010). Effect of ultrasound treatment on fruit decay and quality maintenance in strawberry after harvest. Food Control.

[4] Cheng X.-F., Zhang M., Adhikari B. (2014). Changes in quality attributes of strawberry purees processed by power ultrasound or thermal treatments. Food Sci. Technol. Res..

[5] Yu Y., Xu Y., Wu J., Xiao G., Fu M., Zhang Y. (2014). Effect of ultra-high pressure homogenisation processing on phenolic compounds, antioxidant capacity and anti-glucosidase of mulberry juice. Food Chem..

[6] Anese M., Mirolo G., Beraldo P., Lippe G. (2013). Effect of ultrasound treatments of tomato pulp on microstructure and lycopene in vitro bioaccessibility. Food Chem..

[7] Valdramidis V.P., Cullen P.J., Tiwari B.K., O’Donnell C.P. (2010). Quantitative modelling approaches for ascorbic acid degradation and non-enzymatic browning of orange juice during ultrasound processing. J. Food Eng..

[8] Piyasena P., Mohareb E., McKellar R.C. (2003). Inactivation of microbes using ultrasound: A review. Int. J. Food Microbiol..

[9] Zenker M., Heinz V., Knorr D. (2003). Application of ultrasound-assisted thermal processing for preservation and quality retention of liquid foods. J. Food Prot..

[10] Pingret D., Fabiano-Tixier A.S., Chemat F. (2013). Degradation during application of ultrasound in food processing: A review. Food Control.

[11] Nikkhah E., Khayamy M., Heidari R., Jamee R. (2007). Effect of sugar treatment on stability of anthocyanin pigments in berries. J. Biol. Sci..

[12] Cevallos-Casals B.A., Cisneros-Zevallos L. (2004). Stability of anthocyanin-based aqueous extracts of andean purple corn and red-fleshed sweet potato compared to synthetic and natural colorants. Food Chem..

[13] Tiwari B.K., O’Donnell C.P., Patras A., Cullen P.J. (2008). Anthocyanin and ascorbic acid degradation in sonicated strawberry juice. J. Agric. Food Chem..

[14] Chen F., Sun Y., Zhao G., Liao X., Hu X., Wu J., Wang Z. (2007). Optimization of ultrasound-assisted extraction of anthocyanins in red raspberries and identification of anthocyanins in extract using high-performance liquid chromatography-mass spectrometry. Ultrason. Sonochem..

[15] Fossen T., Rayyan S., Andersen O.M. (2004). Dimeric anthocyanins from strawberry (*Fragaria ananassa*) consisting of pelargonidin 3-glucoside covalently linked to four flavan-3-ols. Phytochemistry.

[16] Abdel-Aal E.S.M., Young J.C., Rabalski I. (2006). Anthocyanin composition in black, blue, pink, purple, and red cereal grains. J. Agric. Food Chem..

[17] Cabrita L., Fossen T., Andersen Ø.M. (2000). Colour and stability of the six common anthocyanidin 3-glucosides in aqueous solutions. Food Chem..

[18] Zhang Y., Sun J., Hu X., Liao X. (2010). Spectral alteration and degradation of cyanidin-3-glucoside exposed to pulsed electric field. J. Agric. Food Chem..

[19] Furtado P., Figueiredo P., Neves H.C.D., Pina F. (1993). Photochemical and thermal degradation of anthocyanidins. J. Photochem. Photobiol. A Chem..

[20] Kırca A., Cemeroğlu B. (2003). Degradation kinetics of anthocyanins in blood orange juice and concentrate. Food Chem..

[21] Vercet A., Lopez P., Burgos J. (1998). Free radical production by manothermosonication. Ultrasonics.

[22] Nayak B., Berrios de J., Powers J.R., Tang J. (2011). Thermal degradation of anthocyanins from purple potato (cv. *Purple majesty*) and impact on antioxidant capacity. J. Agric. Food Chem..

[23] Mercali G.D., Jaeschke D.P., Tessaro I.C., Marczak L.D. (2013). Degradation kinetics of anthocyanins in acerola pulp: Comparison between ohmic and conventional heat treatment. Food Chem..

[24] Sui X., Dong X., Zhou W. (2014). Combined effect of ph and high temperature on the stability and antioxidant capacity of two anthocyanins in aqueous solution. Food Chem..

[25] Yue X., Xu Z. (2008). Changes of anthocyanins, anthocyanidins, and antioxidant activity in bilberry extract during dry heating. J. Food Sci..

[26] Andersen Ø.M. (2002). Anthocyanins. Encyclopedia of Life Sciences.

[27] Brouillard R., Markakis P. (1982). Anthocyanins as Food Colors.

[28] Sun J., Bai W., Zhang Y., Liao X., Hu X. (2011). Identification of degradation pathways and products of cyanidin-3-sophoroside exposed to pulsed electric field. Food Chem..

[29] Sroka Z., Cisowski W. (2003). Hydrogen peroxide scavenging, antioxidant and anti-radical activity of some phenolic acids. Food Chem. Toxicol..

[30] Rice-Evans C.A., Miller N.J., Paganga G. (1996). Structure-antioxidant activity relationships of flavonoids and phenolic acids. Free Radic. Biol. Med..

[31] Li W., Pickard M.D., Beta T. (2007). Effect of thermal processing on antioxidant properties of purple wheat bran. Food Chem..

[32] Zhang Y. (2007). Extraction of Raspberry Anthocyanins Assisted by Pulsed Electric Field. Ph.D. Thesis.

[33] Lopes P., Richard T., Saucier C., Teissedre P.L., Monti J.P., Glories Y. (2007). Anthocyanone a: A quinone methide derivative resulting from malvidin 3-*O*-glucoside degradation. J. Agric. Food Chem..

[34] Floros J.D., Liang H. (1994). Acoustically assisted diffusion through membranes and biomaterials. Food Toconol..

[35] Riesz P., Berdahl D., Christman C.L. (1986). Free radical generation by ultrasound in aqueous and nonaqueous solutions. Environ. Health Perspect..

[36] Kanthale P., Ashokkumar M., Grieser F. (2008). Sonoluminescence, sonochemistry (H_2_O_2_ yield) and bubble dynamics: Frequency and power effects. Ultrason. Sonochem..

[37] Eren Z. (2012). Degradation of an azo dye with homogeneous and heterogeneous catalysts by sonophotolysis. CLEAN—Soil Air Water.

[38] Ruenroengklin N., Yang B., Lin H., Chen F., Jiang Y. (2009). Degradation of anthocyanin from litchi fruit pericarp by H_2_O_2_ and hydroxyl radical. Food Chem..

[39] K De A., Chaudhuri B., Bhattacharjee S. (1999). A kinetic study of the oxidation of phenol, o-chlorophenol and catechol by hydrogen peroxide between 298 K and 333 K: The effect of ph, temperature and ratio of oxidant to substrate. J. Chem. Technol. Biotechnol..

[40] Sun J., Cao X., Bai w., Liao X., Hu X. (2010). Comparative analyses of copigmentation of cyanidin 3-glucoside and cyanidin 3-sophoroside from red raspberry fruits. Food Chem..

[41] Freinbichler W., Colivicchi M.A., Fattori M., Ballini C., Tipton K.F., Linert W., Della Corte L. (2008). Validation of a robust and sensitive method for detecting hydroxyl radical formation together with evoked neurotransmitter release in brain microdialysis. J. Neurochem..

[42] Deng J.J., Cheng J.J., Liao X.Y., Zhang T., Leng X., Zhao G. (2010). Comparative Study on Iron Release from Soybean (*Glycine max*) Seed Ferritin Induced by Anthocyanins and Ascorbate. J. Agric. Food Chem..

[43] Aljadi A.M., Kamaruddin M.Y. (2004). Evaluation of the phenolic contents and antioxidant capacities of two malaysian floral honeys. Food Chem..

[44] Du G., Li M., Ma F., Dong L. (2009). Antioxidant capacity and the relationship with polyphenol and vitamin c in actinidia fruits. Food Chem..

